# An Incidental Finding of Endocervical Melanosis in a Polymyomatous Uterus

**DOI:** 10.7759/cureus.41969

**Published:** 2023-07-16

**Authors:** Nada Akouh, Nassira Karich, Anass Haloui, Amal Bennani

**Affiliations:** 1 Pathology Department, Mohammed VI University Hospital, Faculty of Medicine and Pharmacy of Oujda, Mohammed I University, Oujda, MAR

**Keywords:** rare entity, histological and immunohistochemical confirmation, incidental finding, uterine cervix, melanocytic benign lesion

## Abstract

Melanocytic lesions, whether benign or malignant, are extremely rare in the cervix and, more particularly, in the endocervical mucosa.

Cervical melanosis is a benign entity, most often discovered by incidental findings on a histological study of a surgical specimen resected for another reason. The microscopic examination allows the diagnosis with certainty after ruling out any potential malignancies.

The etiopathogenesis remains poorly understood; however, a number of theories have been put forward, such as excessive migration of pigmented cells from the neural crest, trauma, or chronic irritation situations.

We report the case of a 40-year-old female patient followed in the gynecology department for a polymyomatous uterus. She underwent a total hysterectomy. The histological and immunohistochemical examinations concluded an incidental finding of cervical melanosis lesions associated with leiomyomas.

## Introduction

Melanosis lesions are benign and can affect the uterine cervix [1] such as, in our case, the larynx [2], colon [3], small intestine (jejunum) [4], peritoneum [5], vagina [6], or many other organs.

Endocervical melanosis is a very rare entity with an incidence of 0.05% [7], which is most frequently discovered incidentally in histopathological studies and well analysed so as not to miss the diagnosis of a malignant melanocytic lesion [1]. Microscopic examination is the diagnosis of certainty and shows melanin pigment deposits in the chorion in endocervical localisation and generally hyperpigmentation of the basal layer in case of exocervical localisation [1].

This case report has a double interest: The rarity of the lesion will enable us to review the situation regarding this entity, after ruling out other differential diagnoses, essentially malignant melanic lesions, which are highly aggressive.

## Case presentation

We describe the case of a 40-year-old female patient who was seen in gynecology for well-defined, confluent uterine lesions very suggestive of myomas. A total hysterectomy was done through laparotomy. The specimen was processed for histopathological study. Macro- and microscopic examination of the total hysterectomy specimen confirmed the diagnosis of uterine leiomyomas. The uterine cervix was free of macroscopically detectable lesions.

**Figure 1:**
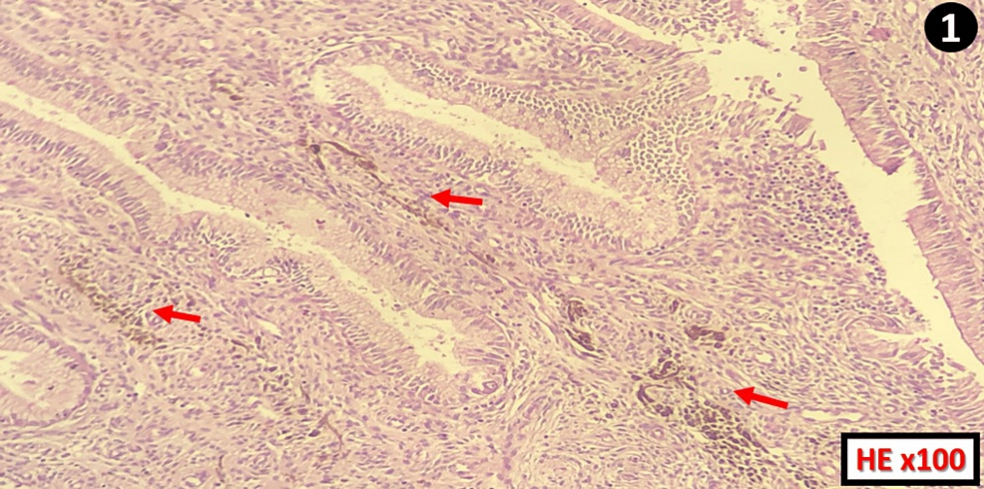
Histological section of an endocervical mucosa made of regular glands and showing melanosis lesions (red arrows) in the chorion HE: hematoxylin and eosin stain

Microscopic examination of the cervix, after staining with hematoxylin and eosin, revealed an endocervical mucosa lined by a regular mucosecretory columnar epithelium, overlying a chorion containing numerous round, ovoid, or spindle-shaped cells, which contained brownish intracytoplasmic pigments without showing cytonuclear atypia or mitosis (Figure1).

**Figure 2:**
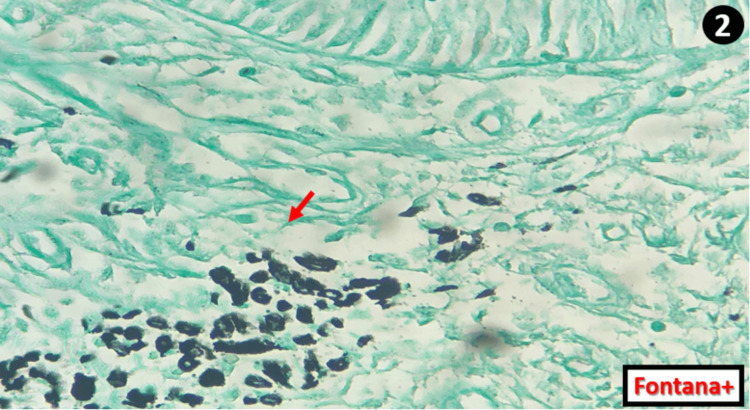
Microphotograph showing the positivity of the intracytoplasmic deposits (red arrow) to Fontana staining confirming their melanic nature

The exocervix consisted of a regular squamous epithelium showing no architectural organisation or signs of HPV infection, overlying a chorion with congestive remodeling and a few scattered lymphocytes and plasma cells. To confirm the highly suspicious melanocytic nature of the intracytoplasmic pigments at the endocervical mucosa, a Fontana staining was performed and came back positive at the level of these deposits (Figure 2).

**Figure 3:**
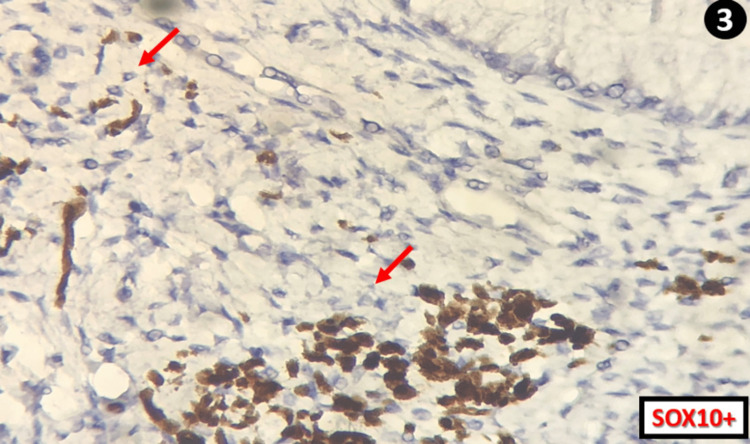
Cells containing the brownish deposits on the HE stain are positive (red arrows) for SOX10 confirming cervical melanosis SOX10: SRY-box transcription factor 10

An immunohistochemical study was also done with SRY-box transcription factor 10 (SOX10) antibody and was also positive (Figure 3).

In light of all these data, the diagnosis of cervical melanosis was retained.

## Discussion

Particularly when it occurs in the endocervix, cervical melanosis is a very uncommon benign disease. In cases of endocervical localisation, it is characterised by localised or diffuse melanin pigment deposits in the chorion. However, in cases of exocervical localisation, it is characterised by hyperpigmentation of the basal layer of the epithelium, with or without accompanying basal melanocytes [1]. The patient with this entity is usually clinically asymptomatic but may present macroscopically with a hyperpigmented lesion, on the speculum, whose diagnosis can only be certain after histological examination [1].

It must be highlighted that any hyperpigmented lesion must be biopsied and histopathologically examined to rule out a malignant neoplasm, especially a mucosal melanoma, which is in turn rarely observed [1].

The pathogenesis of this entity remains poorly elucidated; however, the most widespread theory explains the presence of melanosis lesions at the cervical level as well as at other locations (laryngeal [2], colonic [3], jejunal [4], peritoneal [5], and vaginal [6]), by the fact of the excessive migration of pigmented cells from the neural crest [8]. It has also been related, at the uterine cervix, to chronic irritation situations, frequently as a result of colpocele, falling within the framework of metaplastic changes following chronic inflammation cascades [9]. When these lesions have already been macroscopically identified, they pose a real clinical diagnostic problem, with an extremely wide spectrum of differential diagnoses, ranging from benign lesions, notably nevus with all its variants and seborrheic keratosis, to precursor lesions of malignancy or malignant lesions, notably actinic keratosis, Bowen’s disease, melanoma, squamous cell carcinoma, etc. [6]. All this confirms the necessity and the essential place of histopathology, which will confirm the final diagnosis.

Histologically, it is a matter of intracytoplasmic melanin pigment deposits in histiocytes and melanocytes located in the endocervical chorion. These cells do not show cytonuclear atypia or mitosis, with the absence of ulceration and necrosis. Special staining and/or immunohistochemical study is mandatory to confirm the melanocytic nature of these pigments. If available, electron microscopy is always beneficial [8].

The particularity of our case lies in the rarity of endocervical melanosis, especially since, to our knowledge, this is the third case discovered incidentally during the management of a polymyomatous uterus [8].

## Conclusions

Cervical melanosis lesions are usually asymptomatic and are discovered incidentally on a surgical specimen resected for tumoral or other reasons. When a hyperpigmented lesion is observed clinically, several differential diagnoses arise, and histopathological examination is required. When these patients are followed up after pathological confirmation, no progression to malignancy has been observed.

However, it is important to point out that the etiopathogenesis of these lesions is not clearly studied due to the rarity of the reported cases, and a well-codified follow-up protocol must be programmed.
